# The Dream Experience and Its Relationship with Morning Mood in Adolescents Hospitalized after a Suicide Attempt

**DOI:** 10.3390/brainsci14080804

**Published:** 2024-08-10

**Authors:** Emma Desjardins, Lina Gaber, Emily Larkin, Antoine Benoit, Addo Boafo, Joseph De Koninck

**Affiliations:** 1 School of Psychology, University of Ottawa, 136 Jean-Jacques Lussier, Ottawa, ON K1N 6N5, Canada; edesj086@uottawa.ca (E.D.); lgabe060@uottawa.ca (L.G.); elark042@uottawa.ca (E.L.); abeno099@uottawa.ca (A.B.); 2 Children’s Hospital of Eastern Ontario, 401 Smyth Road, Ottawa, ON K1H 8L1, Canada; aboafo@cheo.on.ca

**Keywords:** nightmares, dreams, mood, suicide, adolescents

## Abstract

Suicidality in adolescents has been associated with emotional distress, stressful life events, relationship issues, and nightmares to name a few. This study explored the actual dream content and the mood at pre-sleep, during a reported dream, and in the morning in 33 adolescents admitted to the hospital on account of a suicide attempt. In all aspects, hospitalized adolescents were compared to 33 matched adolescents who had followed the same protocol. In accordance with the Continuity and the Threat Simulation theories of dream formation, it was hypothesized that the waking-life experiences of suicidal adolescents would transpire in both dream mood and content as well as in the frequency of nightmares. Dreams were analyzed by independent judges using traditional dream content scales, including for the presence of negative and destructive themes and types of interpersonal relationships. As predicted, more suicidal adolescents experienced frequent nightmares, which was significant. A higher negative mood at pre-sleep, within dreams, and at post-sleep was also observed. Furthermore, their dreams contained a higher prevalence of destructive themes and failures, as well as self-directed and death-resulting aggressions. Regression analyses indicated that morning mood was most accurately predicted by positive and negative dream mood in the normative adolescents, whereas only negative dream mood appeared to predict subsequent waking affect in suicidal participants. Our results underline the valuable potential of implementing nightmare-reducing therapies in the presence of suicidal adolescents who suffer from frequent nightmares.

## 1. Introduction

Suicide is a major concern and consistently ranks among the top causes of death for adolescents [1]. For example, Canada reports it as the second-leading cause of death for this age group [2]. In fact, about 90% of Canadian adolescent psychiatric inpatients display high-risk suicide ideation and behaviour [3]. It is thus crucial to develop identification, prevention, and intervention strategies to reduce this risk, and conduct further research to gain a better understanding of the surrounding factors, more specifically, those related to sleep [4].

Previous research has found significant associations between sleep disturbance and the risk of suicidality [5,6,7,8]. In order to more precisely study this phenomenon in real-life situations, we have initiated a series of studies with adolescents admitted to our local hospital psychiatric clinic following a suicide attempt who were willing to share dreams. Relevant to the current report, we have now studied a cohort with full polysomnography and event-related potentials (ERPs) [9,10]. 

Compared to a reference group, they had REM sleep latency abnormalities, shallower sleep, high REM density, and late night slow-wave sleep reduction [9]. ERP measures revealed poorer inhibition in response to emotional stimuli that correlated with shorter REM sleep latency, higher REM sleep, and more frequent nocturnal awakenings [10].

The next step is to explore the dreaming experience of this clinical group again when in crisis. Looking at the dreaming literature, a first observation is that several studies have shown that disturbing dreams, including nightmares and bad dreams, are also associated with a higher risk of suicidality [11,12,13,14,15]. More recent studies have confirmed that experiencing frequent nightmares during the COVID-19 pandemic increased the probability of suicidal ideation [16]. In the current research, we aim to focus beyond nightmares on the actual dream content of our clinical group. Indeed, in a previous study, we observed that frequent nightmare sufferers experience an overall significant difference in the presence of fear and anxiety in their day-to-day dreams that is combined with significantly more negative mood and higher stress levels before and after sleep [17]. 

Looking at the relevant literature, researchers have extensively studied the connection between waking life and dream content. The Continuity Theory (CT) suggests that daytime events, cognitions, and emotions are reflected in dreams in a distorted and selective way [18,19,20]. Further, mundane activities such as reading and writing are seldom represented in dreams, while emotional experiences like stress are preferentially incorporated [20,21,22,23]. Indeed, studies have consistently observed that dreams tend to be more negative than waking experiences, containing more negative emotions, interactions, and outcomes [24]. One of the most well-known studies regarding the negative tone of dream content was the American normative study from Hall and Van de Castle (1966) [25], which has since been reverified and reconfirmed to be present in other samples (more recently for example by Dale et al. 2016) [26]. 

One of the earliest clinical studies of dreams in suicidal patients is that of Gutheil (1948) [27], in which it was determined that dreams not only indicate a patient’s intentions, but typically draw notion to a deeper mental mechanism involved. Gutheil also remarked that as suicide risk increases, dreams representing suicide or death are perceived more positively as a satisfying resolution to a painful situation or conflict [27]. 

Subsequent studies have found that the dreams of suicidal patients contain a high representation of aggressive, violent, and destructive themes and materials relating to death and dying [28,29,30]. Results from Firth et al. (1986) [31] found that although this was true, these destructive themes were most common in patients who were already more aggressive, impulsive, and severely depressed, demonstrating that the link between these dream themes and suicide attempts was indirect, as depression among patients could be attributed to the appearance of the death theme in dreams.

Further research has attempted to determine why negative dream content may be preferentially incorporated in suicidal patient’s dreams. For instance, it has been observed that patients who reported lower levels of psychological well-being tended to have dreams that contained more aggressive than friendly interactions, more negative than positive emotions, and more failures and misfortunes than successes and good fortunes [32]. 

Aǧargün et al. (1998) [12] explored the relationship between recurring and fearful dreams and suicidal tendencies in patients with major depression. Results showed that patients with repetitive nightmares, women specifically, had a higher mean score on the Schedule for Affective Disorders suicide subscale (SADS) than others. A follow-up study also showed a significant correlation between the incidence of nightmares and suicide risk [33]. Recent research has also linked frequent nightmares and depressive symptoms in adolescents. This association appeared to be fully mediated by the distress experienced by individuals due to their recurring nightmares [34].

Given that nightmares and waking emotional distress are linked with suicidality, it is not surprising that individuals with suicidal tendencies present both a predominance of negative affect during waking life and greater difficulty with emotion regulation [10,35,36,37,38,39]. 

Consistent with the CT, it is thus of interest to explore how other waking elements translate into the dreaming experience. Studies have demonstrated that family members are frequently represented characters in children’s dreams. However, according to Foulkes (1982) [40], a general decline has been observed in dreaming about family members in adolescents between 13 and 15 years old [41,42]. However, using a cluster analysis, one study identified family relationships have a typical episode in the dreams of adolescents and young adults [43]. Additionally, Nöltner and Schredl (2023) [44] examined the frequency of core family members in dreams of students and their interactions with the dreamer. This study revealed that about twenty-one percent of the student’s dreams included the mother, the father, or the parent unit as characters [44]. Furthermore, research has also found rejecting-neglecting parenting and low levels of parental care to be significantly associated with youth suicide attempts [45,46]. Using the Parental Acceptance-Rejection Questionnaires (PARQs), one study determined that adolescents who had attempted suicide tended to perceive their parents as more aggressive, neglecting, rejecting, and cold [47]. There is thus a gap in the literature as to how parental rejection and neglect might be translated into the dream experience of adolescents.

To further discuss the relationship between waking and dreaming, the Threat Simulation Theory (TST) postulates that dreaming has evolved as an adaptive survival mechanism to simulate threats drawn from waking-life experiences [48]. In a modern context, individuals who have a more active threat simulation system (TSS) (i.e., traumatized children) have been shown to report a higher number of and more severe dream threats [49]. 

Additionally, the frequency and severity of oneiric threats have been associated with reporting waking-life threatening events, daily stress levels, pre-sleep negative emotions, and recently the presence of COVID-19 pandemic references [50,51,52]. Evidence therefore suggests that the menacing qualities of dreams are influenced by emotional waking-life experiences. The link, however, with the representation of threats in dreams and its value in subsequent adaptation has so far not been established if not ignored, relegating the TST to a dream formation theory.

Moving on to emotions, according to Özdemir et al. (2017) [53], suicide attempters demonstrate greater difficulty in emotion regulation compared to non-attempters as demonstrated by higher scores across all subscales of the Difficulties in Emotion Regulation Scale. This leads to the Emotion Regulation Theory (ERT) of dreaming, which postulates that the purpose of dreams is to regulate emotions by providing a secure and controlled setting for processing emotional experiences from wakefulness, thereby reducing the intensity of negative emotions, like anxiety and fear [54,55,56,57,58]. None of the previous studies, however, have examined the relationship between dream content itself and morning psychological state or mood in order to test this notion.

Of further interest is the phenomenon known as the positivity bias, which has been observed in many studies, wherein individuals evaluating their own emotions in dreams tend to perceive them as more positive than independent judges who analyze their dream narratives [54,59,60,61,62,63]. Some have suggested that the discrepancy between the literal content of the dream narrative and the dreamer’s subjective emotional experience contributes to the process of mood regulation [54]. Since most dreams that are recalled in the morning arise from REM sleep, it has been proposed that the muscle atonia associated with this phase of sleep may assist in desensitizing dreamers from the negative dream content [64].

While the literature so far provides very valuable information on dream content and suicidality, each study has focused on specific components such as dream themes and content, the fearful quality of dreams, and the frequency of nightmares. There is need to conduct a more complete analysis of the dream experience of suicidal adolescents.

The present study aims to contribute to this need by focusing on the dreams of a cohort of adolescents who attempted suicide. These participants were invited to answer a sleep questionnaire covering sleep habits, dream recall, and frequency of nightmares, and report their mood on one night before going to bed (pre-sleep) and upon waking up (post-sleep), and retrospectively write their dreams in the morning from the same night along with their dream mood. This allowed us to examine their dreams and determine the presence and frequency of specific themes in their content compared with a normative sample of adolescents that used the same method of dream collection, except that it was applied in the hospital setting. As established in previous studies, it was expected that the clinical participants would report experiencing a higher frequency of nightmares than observed in normative studies. Based on the Continuity hypothesis, it was further predicted that the participants who had previously attempted suicide would experience dreams that incorporate their waking-life concerns and suicidal ideations, with a greater occurrence of destructive themes, reduced positive social interactions, and a perception of parental neglect and rejection. Additionally, in line with the Threat Simulation Theory, it was expected that these participants would experience more oneiric threats. Regarding mood, both the Continuity hypothesis and TST would predict a positive correlation between pre-sleep mood and dream mood. For instance, the pre-sleep negative experience (i.e., on the previous day) would be expected to result in a more negative mood in the dream. Finally, in the context of the MRT, our objective was to examine the relationship between morning mood, dream mood, and pre-sleep mood. We also sought to determine whether the latter two variables were predictors of morning mood.

## 2. Methods

### 2.1. Participants

The participants were recruited upon admission at the Children’s Hospital of Eastern Ontario (CHEO) inpatient psychiatry unit on account of a suicide attempt within a month of the current admission. All patients admitted to the inpatient psychiatry unit from 1 January 2022 to 30 June 2023 who met the eligibility criteria above were approached. There were 50 of them. Only participants who were willing and able to provide informed consent to take part in the study were included. Eleven declined to participate. Thirty-nine consented to take part in the study and completed it without dropping out. We do not have information as to how the group of 11 compares with the group of 39 as we did not have consent to review their information. Six participants were excluded from the analyses because they did not recall a dream. The suicide attempt group was composed of 33 anglophone adolescents (31 biological females and 2 biological males) between the ages of 13 and 17. It is noted that participants were grouped according to sex assigned at birth. Amongst the biological females, ten participants identified as transgender males, and one as a non-binary individual, and, in the biological males, one identified as a transgender female.

It should be noted that the high proportion of female participants is congruent with the typical observation that females are admitted to inpatient settings more frequently than male counterparts [65]. Indeed, females are more likely than males to experience emotional or sexual abuse, exhibit higher levels of anxiety and depressive symptoms, and have a greater risk for suicidal ideation and attempts [65]. This disparity can also be attributed to the fact that while male adolescents exhibit a higher suicide rate, ranging from twice to four times that of their female counterparts, females display a greater frequency of suicidal attempts, ranging from three to nine times that of males [66,67]. 

Comparator group participants were drawn from a normative study of Canadian dreams conducted at the University of Ottawa Sleep Laboratory; the data for this study were collected from 2004 to 2017. This normative study contains dream reports and surveys from Canadian males and females from the Ottawa–Gatineau area, both anglophones and francophones, from the ages of 12 to 85, divided into five age groups of approximatively 200 per group. It was used in several studies of the ontogenesis of dreams across age groups [68,69]. Each participant completed a daily recording of the day’s events for 10 days or until two dreams were reported. Given the high stability of the dream norms over time, even within cultures, several studies of special populations have used normative data as a benchmark when no control groups were feasible (i.e., Sabourin et al., 2018) [70]. The protocol for dream collections and mood recordings was comparable to that of the normative study, with the exception of the hospital setting, which required consideration. In total, 33 adolescents (31 biological females and 2 biological males; 28 anglophones and 5 francophones) between the ages of 13 and 17 were selected from this normative study to match the mean age and biological sex of the suicide attempt participants. This selection was made according to an inclusion criterion of at least one reported dream from one night, with a dream length of more than 40 words, which is within the norm in dream research to ensure sufficient content. Moreover, the word count between the groups was comparable. No participants from the normative study identified as transgender. No consideration was made of nightmare frequency and actual dream narrative.

### 2.2. Procedure

The 33 adolescents in the suicide attempt group accepted to write their dreams for one night during their stay at the hospital. Patients were invited to participate in the study by a nurse on the ward, and after a verbal agreement, parents and youth signed the consent forms. Then, a psychiatrist interviewed the participants and carried out a diagnostic intake interview and the morning dream report. 

Participants completed an adapted version of the Dream Diary questionnaire from the normative study which is a general questionnaire that explores the sources of dreams, sleep habits, ability to recall dreams, and the influence of dreams on waking life. They rated their mood prior to sleep, upon awakening in the morning, and their dream mood retrospectively. The dream narratives were analyzed by two independent judges for the presence of destructive themes by using Firth’s scale (1986), content themes by using specific categories from the Hall and Van de Castle (HVDC) system of quantitative content analysis (1966), parental rejection by using the PARQ short form child version [71], and threats by using an adapted version of Revonsuo and Valli’s Dream threat scale (DTS) (2000) [72]. Their mood, dream content, and general information were compared to those of the 33 normative adolescents matched for age and gender of the comparator group. This project received full approval from the Ethics Committee of the Children’s Hospital of Eastern Ontario (CHEO) in January 2022.

### 2.3. General Information Questionnaire

The purpose of this questionnaire was to collect information regarding the participants’ demographics (i.e., age, maternal language, sex, marital status, education, employment, and medications), sleep habits (usual sleep schedule, subjective insomnia, and daytime naps), and dream recall (frequency of dreams and nightmares). The frequency of nightmares was determined on a scale of one to six; each number was assigned to a possible frequency range (1—less than one a month; 2—approximately once a month; 3—approximately once per two weeks; 4—approximately one a week; 5—many times a week; 6—almost every night). It should be noted that data regarding sleep habits are not included in this report, as previous cohorts have already been studied and a separate report is currently in preparation. This new report will take into account the time of day or night of the suicide attempt in order to explore the potential influence of circadian rhythms and sleep duration.

### 2.4. Dream Journal

Upon awakening, the participants documented their recalled dreams, providing as much detail as possible to describe the dream’s locations, events, characters, interactions, activities, feelings, and emotions. They also reported their mood both in the evening (prior to sleep), in the morning (after waking up), and, retrospectively, their dream mood. A list of different emotions was rated, and the mood was reported on a Likert scale from 0 to 3, 0 being not at all and 3 a lot. One positive emotion item (Happy) and one negative emotion item (Sad) were used for the temporal analysis of mood and positivity bias.

### 2.5. Destructive Themes

For this study, we selected relevant themes from Firth’s scale (1986) [31], including death [30], destructive or violent hostility [30], separation or loss [73], helplessness [25], masochism [30], realistic versus unrealistic content [73], and hostility [74]. Each theme was evaluated for its presence or absence in the dream narrative and assigned a score of either 1 or 0.

### 2.6. Hall and Van de Castle’s System of Content Analysis

The HDVC instrument is a frequently used and well-validated dream content rating system [75]. Both judges had previously received extensive training in the HDVC coding system and intercoder reliability was established (correlation of 0.80, percentage of perfect match was 92%, *t*-test proved no significant difference between ratings (*t*(2203) = −0.079, *p* > 0.05)). Participants’ dreams were coded for the following categories of the HVDC system of quantitative content analysis: characters, aggression, friendliness, sexuality, success and failure, good fortune and misfortune, and emotions. 

### 2.7. Dream Threat Scale

The methodology employed in this study was based on the approach utilized by Bradshaw et al. (2016) and replicated for the purposes of this research [51]. Threats were categorized following the different levels of threats’ severity established by the DTS: 1.: Life-threatening event, 2.: Socially, psychologically, or financially severe threat, 3.: Physically severe threat, and 4.: Minor threat. The threatening intensity of the dreams was assessed using a 0 to 3 scale, where a score of 3 corresponded to the presence of at least one type 1 (life-threatening) threat as defined by the DTS. A score of 2 indicated the presence of at least one type 2 or 3 (physical or social/psychological/financial) threat, while a score of 1 indicated the presence of at least one type 4 (minor) threat. A score of 0 represented the lowest level of threatening intensity, indicating an absence of threats in the dream content.

### 2.8. Parental Acceptance-Rejection Questionnaire Short Form

The PARQ child version short form was used to determine the dreamer’s perception of parental acceptance-rejection based on the presence of one or both parents (mother and/or father) in the dream narrative. In waking-life research, the PARQ is a self-report instrument, but in this instance of dream content analysis, a judge has used this instrument based on the parental acceptance-rejection reported by the dreamer in the dream narrative. This methodology is derived from previous dream research that used the Eysenck personality inventory to determine if the waking personality of the dreamer has a compensatory or continuous relationship with his oneiric personality [76]. The lowest possible score of the PARQ short form scale is 24 (revealing maximum perceived acceptance), the highest is 96 (revealing maximum perceived rejection), and the midpoint is 60.

### 2.9. Statistical Analysis

The difference in frequency of nightmares between the groups was investigated using a Chi-squared test. The hypotheses of a higher negative and a lower positive reported mood during the pre-sleep, dream, and post-sleep measures were verified using a repeated measure ANOVA. A multiple regression analysis and bivariate correlation analyses were also performed to examine the relationship between moods at the different time points. The expected greater occurrence of waking-life concerns, destructive themes, and oneiric threats and the prediction of reduced positive social interactions in the dreaming experience of the suicide attempt participants were examined using an independent sample *t*-test. Control for multiple comparisons was applied by dividing the error rate by the number of comparisons and adopting a family-wise error rate.

## 3. Results

### 3.1. Nightmare Frequency

**Figure 1:**
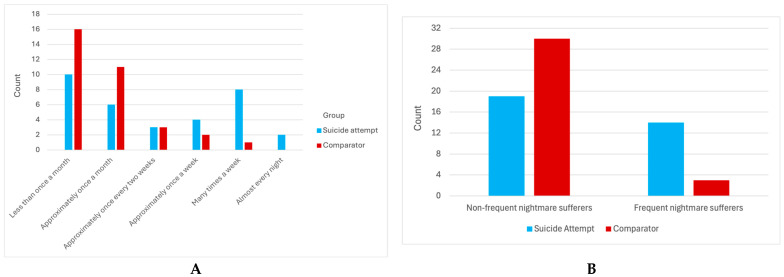
Nightmare frequency according to group. (**A**) illustrates the frequency distribution of participants in each six nightmare frequency ranges (1—less than one a month; 2—approximately once a month; 3—approximately once per two weeks; 4—approximately one a week; 5—many times a week; 6—almost every night) according to group. (**B**) illustrates the frequency distribution of participants in two frequency ranges according to group: those who experience frequent nightmares and those who do not.

Figure 1A illustrates the frequency distribution of participants in each of the six nightmare frequency ranges. The distribution of suicide attempt participants across the items is notably dispersed, with approximately half of them falling into the higher frequency of nightmare items. In contrast, the distribution of the comparator participants is heavily concentrated in the lower frequency of nightmare items. Although there is no frequency criterion for nightmare disorder in the Diagnostic and Statistical Manual of Mental Disorders, Fifth Edition (DSM-5), individuals who experience one or more episodes per week are categorized as presenting a moderate nightmare disorder [77]. Therefore, we established a threshold using this frequency to further differentiate the sample into two subgroups (i.e., frequent nightmare sufferers and non-frequent nightmare sufferers). In that context, a significant difference was observed between the suicide attempt group and the comparator group (Chi-squared = 9.587, *p* = 0.002) (see Figure 1B).

### 3.2. Mood

**Figure 2:**
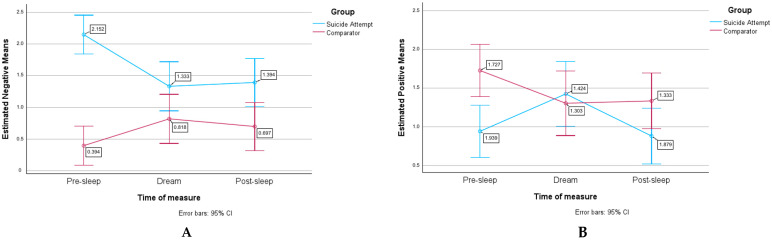
Negative (panel **A**) and positive (panel **B**) moods at pre-sleep, dreaming, and post-sleep in both groups.

The following results are derived from the negative and positive moods (Happy versus Sad) reported in the dream journal. Figure 2 illustrates their variation both in terms of negative (2A) and positive mood (2B) at pre-sleep, dreaming, and post-sleep in both groups.

The suicide attempt group showed a significantly higher combined negative mood (M_SA_ = 1.626) compared to the comparator group (M_C_ = 0.636) while simultaneously reporting significantly lower combined positive mood (M_SA_ = 1.081) compared to the comparator group (M_C_ = 1.455). 

The results of a repeated measure ANOVA showed a significant main effect of time of measure on mood (F(2.63) = 3.874, *p* = 0.023, partial eta square = 0.057), confirming a difference in mood levels between pre-sleep, during dreaming, and post-sleep. There was also a significant interaction between mood and group (F(1.64) = 15.159, *p* < 0.001, partial eta squared = 0.192), indicating a different pattern of mood level variation between the two groups. Although there was no significant interaction between time of measure and group (F(2.128) = 2.427, *p* = 0.092, partial eta squared = 0.037), there was a significant interaction effect between mood, time of measure, and groups (F(2.63) = 12.030, *p* < 0.001, partial eta squared = 0.158); the variation in mood level thus seems to depend on both the group and the time at which the mood was measured; the pattern of mood progression is thus significant and varies between groups.

To investigate the Continuity Theory hypothesis, bivariate correlation analyses were performed on the data of the comparator group (see Table 1) and determined that a positive mood in the evening was strongly and positively correlated with a positive mood during dreaming (*r* = 0.605, *p* < 0.01), and strongly and positively correlated with a positive mood in the morning (*r* = 0.563, *p* < 0.01). Additionally, a positive mood during dreaming was strongly and positively correlated with a positive mood in the morning (*r* = 0.724, *p* < 0.01). A positive mood in the morning was moderately and negatively correlated with a negative mood during dreaming (*r* = −0.463, *p* < 0.01).

Furthermore, fostering a negative mood during dreaming was moderately and positively correlated with a negative mood in the morning (*r* = 0.495, *p* < 0.01).

Bivariate correlation analysis was performed on the data of the suicide attempt group (see Table 2) and determined that a positive mood in the evening was moderately and positively correlated with a positive mood in the morning (*r* = 0.371, *p* < 0.05). Not surprisingly, a positive mood during dreaming was moderately and negatively correlated with a negative mood during dreaming (*r* = −0.405, *p* < 0.05). A positive mood in the morning was moderately and negatively correlated with a negative mood in the evening (*r* = −0.362, *p* < 0.05).

Furthermore, fostering a negative mood in the evening was moderately and positively correlated with negative mood during dreaming (*r* = 0.393, *p* < 0.05) and moderately and positively correlated with negative mood in the morning (*r* = 0.391, *p* < 0.05). A negative mood during dreaming was moderately and positively correlated with a negative mood in the morning (*r* = 0.406, *p* < 0.05).

An objective of this study was to determine whether the pre-sleep and dream mood variables could be used as predictors of morning mood. To this end, a multiple regression analysis was performed. It revealed that among suicide attempt participants (n_SA_ = 33), reporting a positive mood in the evening significantly and positively predicted reporting a positive mood in the morning (β = *0*.316, *p* = 0.036). However, reporting a positive mood during dreaming was not found to be a robust predictor of mood in the morning (β = *0*.195, *p* = 0.212). Moreover, the results indicated that reporting a negative mood during dreaming was a moderate predictor of a negative mood in the morning (β = 0.298, *p* = 0.045) in this group. 

**Figure 3:**
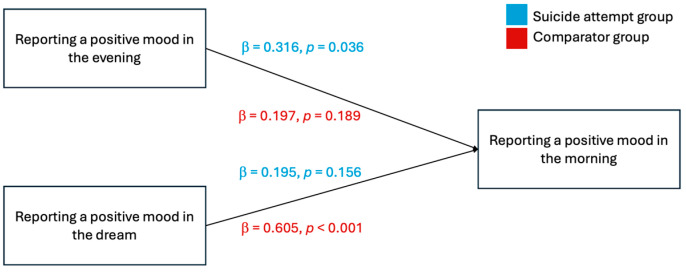
Illustration of a positive mood in the morning predicted by the report of a positive mood in the evening or in the dream.

**Figure 4:**
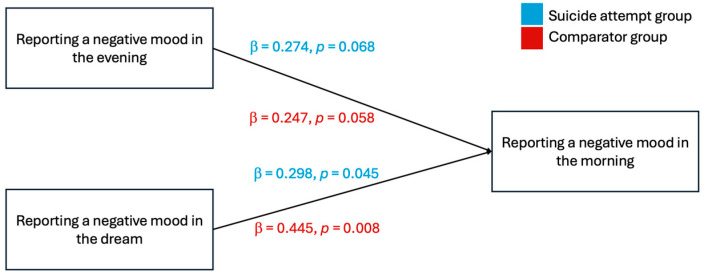
Report of a negative mood in the morning predicted by the report of a negative mood in the evening or in the dream. Notes. Standardized coefficients are presented. The estimates above each line correspond to the estimate for the suicide attempt group, while the estimates presented below each line correspond to the comparator group.

Among the comparator group (n_C_ = 33), reporting a negative mood during dreaming moderately predicted reporting a negative mood in the morning (*β* = 0.445, *p* = 0.008), while a negative mood in the evening failed to predict a negative mood in the morning (*β* = 0.247, *p* = 0.058). Moreover, reporting a positive mood during dreaming had a strong significant positive predicting value of reporting a positive mood in the morning (*β* = 0.605, *p* < 0.001), yet there was no significant relationship between reporting a positive mood in the evening and in the morning (*β* = 0.197, *p* = 0.189) in the comparator group. Results of the multiple regression are illustrated in Figure 3 and Figure 4.

### 3.3. Positivity Bias

The result of an independent sample *t*-test comparing the suicide attempt group’s average self-evaluated dream mood (n_SA_ = 33) to an external judge’s evaluation of the dream mood revealed that there was a significant difference in terms of positive mood between the participant’s self-evaluation and the external judge’s evaluation of the dream mood (*t*(60.520) = 3.427, *p* < 0.001, Cohen’s’ *d* = 0.844). To be more precise, the dream mood was evaluated on average more positively by participants from the suicide attempt group themselves than by an external judge (M_SA_ = 1.424, M_EJ_ = 0.5152). This is consistent with the previous literature on this phenomenon [54].

### 3.4. Dream Content 

Destructive themes. Results from independent sample *t*-tests comparing the comparator group’s dreams (n_SA_ = 33) to our suicide attempt group’s dreams (n_C_ = 33) revealed that there was no significant difference in terms of the presence of destructive themes between the dream content of the comparator group and the suicide attempt group (*t*(64) = 1.487, *p* = 0.071). After applying control for multiple comparisons, the most statistically significant individual theme differences were those of death (*t*(51.450) = 2.530, *p* = 0.007, Cohen’s *d* = 0.623) and masochism (*t*(32) = 3.028, *p* = 0.002, Cohen’s *d* = 0.745) (see Table 3). To be more precise, the dream content of participants from the suicide attempt group featured on average more death (M_SA_ = 0.273, M_C_ = 0.061) and more masochism (M_SA_ = 0.197, M_C_ = 0.00) compared to the participants from the comparator group.

Hall and Van de Castle categories. When comparing the dreams of the comparator group (n_C_ = 33) to those of the suicide attempt group (n_SA_ = 33) using independent sample *t*-tests, we found a significant disparity in the number of self-directed aggressions (*t*(32) = −1.936, *p* = 0.031, Cohen’s *d* = 0.477), friendly interactions (*t*(48.468) = −2.387, *p* = 0.010, Cohen’s’ *d* = −0.588), failures (*t*(49.039)= 2.549, *p* = 0.007, Cohen’s *d* = 0.628), and level of happiness (*t*(45.493)= 2.030, *p* = 0.024, Cohen’s *d* = 0.500) within the dream content (see Table 4). To be more precise, the dream content of participants from the suicide attempt group featured on average more self-directed aggressions (M_SA_ = 0.242, M_C_ = 0.000), fewer friendly interactions (M_SA_ = 0.379, M_C_ = 1.000), more failures (M_SA_ = 0.288, M_C_ = 0.0601), and a higher level of happiness (M_SA_ = 0.333, M_C_ = 0.091) compared to the participants from the comparator group. In the friendliness category, the sharing of pleasant social activities subcategory emerged as the most significant (*t*(32) = −2.667, *p* = 0.006, Cohen’s’ *d* = −0.656). Despite there being no statistically significant difference in the overall quantity of aggressions in the suicide attempt group’s dream content (*t*(64) = −1.168, *p* = 0.123) when compared to the comparator group, there was a significantly higher incidence of death-resulting aggression (type 8 aggression) (*t*(32) = −2.620, *p* = 0.007, Cohen’s’ *d* = 0.645) within this group (M_SA_ = 0.197, M_C_ = 0.000). 

Threats. As illustrated in Table 5, no significant difference in the total quantity of threats between the two groups (n_SA_ = 33, n_C_ = 33) was revealed by the statistical analyses, but a higher quantity of psychological/financial/social type threats (t(57.432)= 2.081, *p* = 0.021, Cohen’s’ d = 0.512) was observed in the suicide attempt group’s dream content (M_SA_ = 0.636, M_C_ = 0.303). The dreams of this group also seemed to demonstrate a higher threatening intensity in terms of psychological and physical integrity (t(64) = 2.029, *p* = 0.023, Cohen’s’ d = 0.500) when compared to those of the comparator group (M_SA_ = 1.727, M_C_ = 1.212).

Parental Acceptance-Rejection Questionnaire short form. Among the 33 suicidal dreams, 14 contained at least one parent character (42% of the dreams), whereas in the comparator group, the presence of parental figures was observed in only 9 out of the 33 dreams (27% of the dreams). Only three dream narratives from the suicide attempt group and one dream narrative from the comparator group included sufficient detailed interactions with a parental character to allow for analysis of the relationship using the PARQ short form. The average score of parental acceptance-rejection in the suicide attempt group was 72.667. As for the only comparative score for parental acceptance-rejection, the result was 45.

### 3.5. Dream Sample

In order to demonstrate how elements from waking life can be incorporated into dreams, we present examples of dreams shared by participants in the suicide attempt group:

“(…) another person I do not really remember who it was, but he was talking about like how his life was like falling apart and I saw him like self-harming a lot and there was a lot of blood and I remember that the blood was like dripping onto snow, below him so it is really easy to see because it was like bright red on the white snow (…).”

“(…) As a result of these emotions I abruptly said I was going to leave. I started packing my suitcase. My mom questioned why I was leaving and I said nobody wants me around that’s why. We had a very verbal and loud argument about it. I remember thinking I hoped they cared enough to tell me to stay but they didn’t. In this situation I felt like a burden to everyone.”

## 4. Discussion

In the context of current theories, and clinical observations and interventions, the main goal of this study was to investigate the mood, frequency of nightmares, and presence and frequency of specific themes in the dreams of suicidal adolescents, as compared to a normative sample of adolescents. We comment here on the implications of the findings.

### 4.1. Nightmare Frequency

First, our findings demonstrate that the suicide attempt group had a greater number of participants who could be considered as frequent nightmare sufferers than those in the comparator group, with nearly fifty percent of the suicide attempt group reporting one or more nightmares a week. This finding is consistent with the existing literature, which identifies that frequent nightmares are a risk factor for suicidality [12,33]. It is important to note that the occurrence of frequent nightmares does not necessarily indicate the presence of a nightmare disorder, as additional DSM-5 criteria must be met [77]. Nevertheless, addressing nightmares may be an important component of suicide prevention and intervention efforts for adolescents.

The literature demonstrates that non-pharmacological interventions, such as Imagery Rehearsal Therapy and lucid dreaming, are effective in reducing nightmares in both children and adolescents [78,79]. For instance, Imagery Rehearsal Therapy has been linked to enhanced sleep quality and reductions in the severity of PTSD symptoms in sexual assault survivors [80]. Nielsen and Levin (2007) proposed the affective network dysfunction model, suggesting that nightmares are caused by a disruption in the adaptive function of fear memory extinction during dreaming [81]. Reducing nightmares could improve stress and emotional distress associated with fear memory and sleep quality, thus acting as a promising preventive strategy for suicide among adolescents who have major depression and experience frequent nightmares.

### 4.2. Mood

Next, as shown by our results, our suicide attempt group demonstrated a significantly higher combined negative mood, and a significantly lower combined positive mood, at night, in dreams, and in the morning, in comparison to the comparator group. This observation is consistent with prior research, and supports that negative affect is strongly associated with an increased risk for suicidality [37,39].

As illustrated in both panels of Figure 2, our results revealed a significant relationship between mood, time, and group, indicating that the mood varied when controlled for the time it was measured and the group it pertained to.

In terms of negative mood, the progression throughout time periods was different between the two groups, showing a reverse pattern. The comparator group exhibited a typical negative mood progression, with a low negative mood level at the pre-sleep measure and an increase in negative emotions during dreaming. Indeed, this pattern is consistent with previous research, wherein participants have typically reported a greater prevalence of negative emotions, or a “negative tone”, in their dreams in comparison to their waking life [24,25,26,82]. This negative tone has been associated with the predominant activation of the amygdala, a brain structure involved in the processing of fear, during rapid eye movement (REM) sleep [24,83]. In our study, however, the suicide attempt group had baseline levels of evening negative mood so high that the dream and post-sleep moods were low in comparison.

It is of value to reflect on what the implication of these findings is in relation to the ERT. In our study, the levels of negative emotions in dreaming and in waking reported by suicide attempt participants differ from the pattern seen in the comparator participants and previous research. This may suggest an alteration in the processing of emotion regulation during dreaming, and reflect the observed difficulties in regulating emotions during wakefulness among individuals with suicidal tendencies [53]. This observation is also consistent with our previous findings regarding disrupted rapid eye movement (REM) sleep and waking emotional processing in a different cohort of the same patient group, as mentioned in our introduction [9,10]. Despite this difference in pattern, the suicide attempt group still reported a lower negative mood at the post-sleep measure than at the pre-sleep measure, indicating some improvement between the two measures of time. This is consistent with previous research that has demonstrated enhanced emotional adjustment, mood, and well-being after intact sleep, particularly REM sleep, even when disrupted [84].

Further, it is of interest to examine the progression of positive mood with the suicide attempt group, who reported lower positive mood levels than the comparator group before and after sleep, but not in the dream. More specifically, in the comparator group, positive mood was higher at pre-sleep, decreased during dreaming, and then remained stable at the post-sleep measure. Suicidal participants started with low positive mood levels at pre-sleep, which increased during dreaming to rise above the level of the comparator group and decreased at the post-sleep measure. A similar observation emerged in the study of Punamäki (1999), where they found that a negative evening mood in children residing in traumatic environments was associated with the report of positive dreams [85]. The author attributed this phenomenon to a model of compensatory dream function, whereas the presence of these positive emotions could be the result of a psychological mechanism that attempts to protect the dreamer’s well-being by providing a relief from a painful reality [85,86,87]. This would align with the disruption-avoidance-adaptation model of dreaming, where disruption facilitates mastery over a stressful stimulus, while avoidance protects the dreamer against this disruption to ultimately allow adaptation [86].

Furthermore, it is interesting to note that the progression of positive mood in the suicide attempt group is similar to the progression of negative mood in the comparator group with an increase in mood during the dream. Also, the progression of negative mood in the suicide attempt group is similar to the progression of positive mood in the comparator group with a decrease in mood during the dream. Thus, it would seem that the progression in mood is reversed according to the valence between the groups. This is also consistent with the disruption-avoidance-adaptation model of dreaming. From this perspective, the observed increase in positivity among adolescents who have attempted suicide could be indicative of avoidance, whereas the increase in negativity among the comparators could be indicative of mastery.

The findings from the bivariate correlation on the comparator group’s mood partially aligned with the CT of dreaming. Specifically, a positive mood in the evening was associated with a positive mood during the dream and in the morning. Indeed, previous studies have demonstrated that emotional experiences and mood states in wakefulness can influence the nature of dreams, as positive emotional experiences tend to lead to more positive and pleasant dreams, whereas negative emotional experiences tend to lead to more negative and unpleasant dreams [88,89,90].

In addition, a positive mood during the dream was strongly linked to a positive mood in the morning and a negative mood during dreaming was moderately associated to a negative mood in the morning. Conversely, a negative mood during the dream was moderately and negatively linked to a positive mood in the morning. These findings are consistent with the discussion by Mallett et al. (2021) proposing that dream experiences can influence subsequent waking affect [91].

The suicide attempt group’s bivariate correlation results were also partially consistent with the CT of dreaming. Specifically, a negative mood in the evening was linked to a negative mood during dreaming and in the morning. Additionally, a negative mood during dreaming was moderately linked with a negative mood in the morning. These findings collectively indicate that the reported negative moods were consistently related ted across the different times of measure.

The multiple linear regression results suggest that both positive and negative dream mood is a predictor of morning mood in the comparator group. This is consistent with the hypothesis that dreaming plays a role in regulating emotions upon waking, as proposed by the ERT [54].

Contrary to the results of the comparator group, the positive dream mood of the suicide attempt group was not found to be an indicator of the morning mood. Instead, only the positive evening mood was found to be an indicator of the morning mood. Nevertheless, a negative dream mood was found to be a moderate predictor of a negative mood in the morning in this group. Once more, the emotion regulation process hypothesized by the ERT may be altered in this group as only the negative dream mood seems to predict the subsequent waking affect.

Based on these preliminary results, it can be hypothesized that there is a tentative compensation effect in dreaming, as evidenced by the increased positive mood. However, this effect does not appear to be sufficient to ameliorate mood in the morning. This supports the recommendation that improving dream experience may improve mood in adolescents at risk for suicidality.

### 4.3. Positivity Bias

Moreover, the suicide attempt group exhibited a discrepancy between the participants’ and the judge’s evaluations regarding the positive mood in the dream. Thus, despite the potential alteration of emotion regulation in dreaming discussed before, this observation demonstrates that the suicide attempt group exhibited a positive bias. Such a bias has been hypothesized to aid in the desensitization process that may occur during dreams [54] and may contribute to the lower post-sleep negative mood observed in the suicide attempt group.

### 4.4. Dream Content

Destructive themes. Our research findings corroborate the conclusions drawn by Firth et al. (1986), in that the occurrence rate of dreams concerning death and dying and featuring the theme of masochism was considerably greater among individuals with suicidal tendencies than in the comparator group [31]. Negative self-concept has previously been associated with suicidal ideation and attempts in youth [92,93,94]. This relationship between negative self-concept and suicidal tendencies may explain why both themes, masochism and death, appeared more frequently in the dreams of suicidal adolescents. Research has shown that helplessness and hopelessness, which are closely related strong predictors of suicidality [95,96]. In a study conducted by Glucksman and Kramer (2017), these factors have also been observed as significant themes in the dream content of clinically depressed potentially suicidal patients [28]. However, in our study, when examined individually, the other destructive themes did not exhibit any notable differences between the two groups. Indeed, the analyzed theme of helplessness did not display a significant difference in frequency between the groups. This could potentially be attributed to the challenge that the judges faced in inferring from the dream narrative whether the dreamer experienced a feeling of helplessness.

### 4.5. Hall and Van de Castle Categories

Friendliness. Positive social relationships, such as social support and friendships with others, tend to be protective factors against suicidal behavior [97,98]. Our finding that suicidal adolescents reported significantly fewer friendly interactions in their dream is consistent with the Continuity Theory of dreaming, in that it mirrors their waking deficit in positive social interactions in their dreams compared to normative adolescents.

Aggression. Self-directed aggressions are any form of aggression from the HVDC scale accomplished by and directed toward the dreamer. This category is very similar in concept to the masochism theme analyzed earlier. Consistent with our prior presented results, the prevalence of self-directed aggression on the HVDC scale confirms the higher frequency of negative self-thoughts or actions in the dream content of the suicide attempt group.

Prior studies have demonstrated that aggressivity can be considered a predictor of suicide attempts or even a feature of suicide capability [99,100]. Our results showed no statistically significant difference in the overall quantity of aggressions between the two groups. However, there was a significantly higher incidence in dreams of death-resulting aggression within the suicide attempt group. This category is established as the highest intensity of aggressive interactions on the HVDC scale. This result could be associated with the prevalence of death-related themes in dream content, thus indicating that not only is death more frequent but also more aggressively portrayed.

Failure. Our analysis indicates that the suicide attempt group had a significantly higher quantity of failures compared to the comparator group. In the context of dream content, “failure” is defined by the HVDC scale as the inability of a character to achieve their desired goal due to personal limitations and inadequacies [25]. This definition can be associated with the negative self-concept, helplessness, and hopelessness discussed earlier. This result can suggest that suicidal dreamers perceive a lack of control, which prevents them from reaching their goals.

Happiness. The higher level of expressed written feelings of happiness in the dream content of the suicide attempt group is consistent with the higher reported positive mood associated with the dream experience in the suicide attempt group when compared to the comparator group, and the increase in positive mood observed from evening to dream in the suicide attempt group, as depicted in Figure 2B. The same elements as discussed in the mood section of this paper in relation to the possibility of a different emotion regulation process during dreaming in the clinical sample could be hypothesized from this result. Also consistent with this observation is the fact that the positivity bias was observed in the suicide attempt group and interpreted as a tentative manifestation of mood regulation. In addition, this combination of positive with negative content has been observed recently by Vallat et al. (2018) [101] and Barbeau et al. (2022b) [54] and interpreted as possible desensitization attempts. However, further replication and investigation of this relationship is necessary, and the utilization of text analysis tools (text mining software) could yield valuable insights on this matter.

### 4.6. Threats

Our findings showed that the number of threats in the dream content of suicidal adolescents was not higher than the one of normative adolescents. Nevertheless, the higher quantity of type 3 threats or psychological/financial/social type threats and a higher threatening intensity in terms of psychological and physical integrity observed in the suicide attempt group’s dream content are consistent with prior research on waking clinical correlates for suicidal ideation; suicide has indeed been closely associated with psychological distress, depression, and higher levels of stress (i.e., traumatic, interpersonal) in adolescents [102,103,104]. Threatening and negative dreams have also been associated with rumination and intrusive thoughts, which are closely related to depression, hopelessness, and suicidality [105,106].

### 4.7. Parental Acceptance-Rejection Questionnaire Short Form

There was a slightly higher frequency of the presence of parental characters in the dreams of suicidal adolescents, with 42% compared to 27% in control participants. The details about the relationship with the parent also seemed higher in the suicide attempt group, as three dream narratives allowed for analysis with the PARQ short form as opposed to one in the comparator group. The average parental-acceptance score was higher in the suicide attempt group than in the comparator group, suggesting that suicidal adolescents conveyed a more rejecting parental image in their dream narrative. This result is consistent with prior research in which suicide attempters tended to perceive their parents to be more rejecting [47]. A potential avenue for further investigation could be to assess the perceived level of parental acceptance-rejection during waking and correlate the result to the inferred one from the dream narrative of suicidal adolescents. Completing the PARQ short form during the day could also trigger parental presence in the dreams of adolescents, as elements from salient or emotional recent waking-life experiences can be integrated into dreams [107,108].

### 4.8. Transgender and Non-Binary Participants

Finally, it is important to note that among the thirty-three suicidal participants in this study, twelve individuals identified as a gender different from that assigned at birth. Specifically, ten participants identified as transgender males, one as a transgender female, and one as non-binary. This percentage is representative of, and in accordance with, the prior established prevalence of suicidality in this population. With estimates ranging from 31% to 64%, transgender individuals exhibit a significantly higher risk for depression and anxiety than the general population [109]. Research shows that transgender adolescents are more likely to report suicide risk than cisgender adolescents [110]. Stigma, LGBT victimization, and gender non-conformity have been associated with self-harm and/or suicidal ideation [111]. There may be differences in dream content between transgender and cisgender individuals in terms of interactions and identity. Conducting a larger study with more participants could yield valuable insights into how gender identity manifests in dream content.

### 4.9. Limitations

#### Several Factors May Influence the Implications of the Results of this Study

Dream recall. Dream recall is known to be imperfect as dream content can be reinterpreted and rationalized upon waking [112]. Dream reports often fail to capture the full scope and intensity of the dream experience. Indeed, dreamers tend to provide minimal details and overlook the grander aspects of their dreams when recounting them. As a result, the experiences described in dream reports can be quite different from the actual dream experience [113]. Furthermore, the situation of hospitalization, the medication, and multiple other factors can affect dream recall [24]. Although the dream diary method is a superior approach to questionnaires, it is still far from ideal [24]. Additionally, the field of brain recording of dream activity is still in its infancy [24].

Convenience sample. The clinical sample for this study was recruited through an emergency hospital inpatient unit on a volunteer basis and represents a convenience sample. Consequently, the conclusions presented in this article should be considered preliminary and may not be representative of a more general population and, in particular, of non-hospitalized adolescents who have attempted suicide. Nevertheless, these findings are highly relevant as they were obtained using the current standard of dream collection and warrant further investigation with larger samples. Finally, as we mentioned earlier, the sleep habit data were not included in this report since they were studied in our previous cohort and are included with a larger sample of circadian and sleep factors as well as timing of suicide attempt.

## 5. Conclusions

Our study provides support for the CT of dreaming, as it unveils that the dreams of suicidal adolescents reflected different aspects of their waking life. These findings align with the emotional dysregulation and distress, negative affect, negative self-concept, parental rejection, lack of social support, and suicidal ideation previously associated with at-risk suicidal adolescents. Our results underline the value in addressing nightmares, and support utilizing intervention techniques and therapies to mitigate the negative experience of dream content in relation to suicidal tendencies. By allowing adolescents who experience frequent nightmares and suicidal ideations to undergo therapies aimed at reducing nightmares, emotional distress could be alleviated, sleep quality could be improved, and overall psychological well-being could be enhanced. The observed association of positivity bias and morning mood suggests that further studies should explore this relation and the empirical value of the ERT in the context of adolescent suicidality. It would also be of interest to investigate the documented emotion regulation value of REM sleep in this population [114].

## Figures and Tables

**Table 1:** Correlation table for positive and negative moods of the comparator group at pre-sleep, dreaming, and post-sleep.

		Positive Mood			Negative Mood		
		Evening	Dreaming	Morning	Evening	Dreaming	Morning
**Positive mood**	Evening	1	0.605 **	0.563 **	−0.398 *	−0.326	−0.224
	Dreaming		1	0.724 **	−0.101	−0.425	−0.209
	Morning			1	−0.120	−0.463 **	−0.455 **
**Negative mood**	Evening				1	0.203	0.337
	Dreaming					1	0.495 **
	Morning						1

**. Correlation is significant at the 0.01 level (2-tailed). *. Correlation is significant at the 0.05 level (2-tailed).

**Table 2:** Correlation table for positive and negative moods of the suicide attempt group at pre-sleep, dreaming, and post-sleep.

		Positive Mood			Negative Mood		
		Evening	Dreaming	Morning	Evening	Dreaming	Morning
**Positive mood**	Evening	1	0.279	0.371 *	−0.553 **	−0.177	−0.196
	Dreaming		1	0.283	−0.133	−0.405 *	−0.316
	Morning			1	−0.362 *	−0.082	−0.188
**Negative mood**	Evening				1	0.393 *	0.391 *
	Dreaming					1	0.406 *
	Morning						1

**. Correlation is significant at the 0.01 level (2-tailed). *. Correlation is significant at the 0.05 level (2-tailed).

**Table 3:** Comparison of destructive themes’ frequency in dream content between groups.

	*t*	*df*	*Significance (One-Sided p)*	*Cohen’s d*
*Death*	2.530	51.450	0.007 **	0.623
*Violent Hostility*	0.593	64	0.278	-
*Separation*	−0.175	64	0.431	-
*Helplessness*	0.542	64	0.295	-
*Masochism*	3.028	32	0.002 **	0.745
*Unrealistic content*	0.000	64	0.500	-
*Hostility*	0.137	64	0.446	-
*Sum*	1.487	64	0.071	-

**. Correlation is significant at the 0.01 level (one-tailed), after control for multiple comparisons.

**Table 4:** Comparison of Hall and Van de Castle categories’ frequency in dream content between groups.

	*t*	*df*	*Significance (One-Sided p)*	*Cohen’s d*
*Characters*	−1.334	64	0.093	-
*Total Aggression* *Death-resulting aggression*	1.168 2.620	64 32	0.123 0.007 **	- 0.645
*Self-directed aggression*	1.936	32	0.031 *	0.477
*Friendliness* *Pleasant activity sharing*	−2.387 −2.667	48.468 32	0.010 * 0.006 **	−0.588 −0.656
*Friendliness towards dreamer*	−1.782	58.428	0.040	−0.439
*Sexuality*	−0.632	64	0.265	-
*Success*	0.436	64	0.424	-
*Failure*	2.549	49.039	0.007 **	0.628
*Misfortune*	0.490	64	0.313	-
*Good Fortune*	−0.149	64	0.441	-
*Negative emotions*	1.445	64	0.077	-
*Happiness*	2.030	45.493	0.024 *	0.500

**. Correlation is significant at the 0.01 level (one-tailed). *. Correlation is significant at the 0.05 level (one-tailed).

**Table 5:** Comparison of threat frequency in dream content between groups.

	*t*	*df*	*Significance (One-Sided p)*	*Cohen’s d*
*Total threats*	0.942	64	0.175	-
*Type 1—Life-threatening*	−0.119	64	0.453	-
*Type 2—Psycho./Financial/Social*	2.081	57.432	0.021 *	0.512
*Type 3—Physical*	0.000	64	0.500	-
*Type 4—Minor*	−0.407	64	0.343	-
*Threatening intensity*	2.029	64	0.023 *	0.500

*. Correlation is significant at the 0.05 level (one-tailed). **. Correlation is significant at the 0.01 level (one-tailed).

## Data Availability

The data base of our manuscript will be available upon request, respecting the confidentiality and the consent of participants in accordance with the ethic approval of the project. Copies of questionnaires are also available.
